# Vacuum Packaging Can Protect Ground Beef Color and Oxidation during Cold Storage

**DOI:** 10.3390/foods13172841

**Published:** 2024-09-07

**Authors:** Gabriela M. Bernardez-Morales, Savannah L. Douglas, Brooks W. Nichols, Ricardo J. Barrazueta-Cordero, Aeriel D. Belk, Terry D. Brandebourg, Tristan M. Reyes, Jason T. Sawyer

**Affiliations:** 1 Department of Animal Sciences, Auburn University, Auburn, AL 36849, USA; gzb0063@auburn.edu (G.M.B.-M.); sld0060@auburn.edu (S.L.D.); bwn0004@auburn.edu (B.W.N.); rzb0143@auburn.edu (R.J.B.-C.); adb0097@auburn.edu (A.D.B.); tdb0006@auburn.edu (T.D.B.); 2 Winpak Ltd., 100 Saulteaux Crescent, Winnipeg, MB R3J 3T3, Canada; tristan.reyes@winpak.com

**Keywords:** ground beef, instrumental color, lipid oxidation, storage period, vacuum packaging

## Abstract

Storing ground beef at frozen temperatures prior to refrigerated display when using thermoforming vacuum packaging is not a common manufacturing practice. However, limited data on thermoforming packaging film and its interaction with meat quality suggests that more information is needed. The current study aimed to identify the influences of thermoforming packaging on the surface color and lipid oxidation of ground beef. Ground beef was portioned into 454 g bricks and packaged into one of three thermoforming films: T1 (150 µ polyethylene/EVOH/polyethylene coextrusion), T2 (175 µ polyethylene /EVOH/polyethylene coextrusion), and T3 (200 µ polyethylene/EVOH/polyethylene coextrusion), stored for 21 days at −20.83 °C (±1.50 °C), and displayed for 42 days at 3.0 °C ± 1.5 °C. There were no statistical differences for the packaging treatment of lipid oxidation (*p* = 0.0744), but oxidation increased throughout storage day (*p* < 0.0001). The main effects of treatment and day resulted in altered (*p* < 0.05) surface lightness (L*), redness (a*), yellowness, hue angle (°), red-to-brown (RTB), and relative myoglobin for met-myoglobin (MET), deoxymyoglobin (DMB), and oxymyoglobin (OMB). Surprisingly, there was an interaction between treatment and day for the calculated relative values of chroma (*p* = 0.0321), Delta E (*p* = 0.0155), and the ratio of a*:b* (*p* < 0.0001). These results indicate that thermoforming vacuum packaging can reduce the rate of deterioration that occurs to ground beef color and the rate of oxidation.

## 1. Introduction

Storing meat products is a vital stage in delivering protein options to the consumer within the retail or food service sector of the U.S. Seldom do manufacturers recommend freezing ground beef during logistical patterns and retail display. Frozen portioned consumer meat products will often deteriorate rapidly if stored frozen prior to refrigerated store display, altering the surface color and compromising consumer purchasing. Despite efforts to stimulate consumer demand for beef, ground beef sales are increasing at a rate of 39.2% of total dollars and representing under half the consumer purchases of retail sales in the U.S. [1,2]. It has been estimated that 53.7% of total ground beef in the retail arena is sold in poly-vinyl chloride packaging, which is well-documented for causing rapid color decline and greater discarding [3]. Recently, ground beef preference has increased consumer purchase rates, and, despite a 1.8% decline in pounds sold during December 2023, ground beef is purchased more often than whole-muscle cuts [3]. Regardless of variability in fresh meat quantities in the U.S., logistical management of perishable meats for consumer products like ground beef can be difficult. No research has evaluated the combination of freezing, thawing, and refrigerated storage for fresh beef during retail consumer presentation.

Meat products sold within the U.S. meat industry may vary in storage duration, surface color, or even flavor profile. Storing meat at frozen temperatures prior to retail offering may curtail the volume of fresh ground beef that is discarded at the retail level when using creative packaging methods [4]. Unfortunately, little is known about the surface color development following the frozen storage of ground beef. Every year, it is estimated that over 1.3 billion pounds of ground beef is manufactured for retail purposes, occupying more shelf space per linear foot in retail stores than any other fresh meat products [2]. Considering the versatility and widespread consumption of ground beef in the U.S., it is often cataloged as the undisputed leader [4]. Therefore, identifying methods for extending storage duration without altering surface color both in-store and at-home for the consumers of ground beef is needed. 

Ground beef leads the progression of packaging technologies within the domain of red meats due to greater food flexibility, creating an easy way to justify such developments compared to meat cuts and other food products [4]. Traditionally, consumers have relied on color as the predominant signal of freshness and quality, often seeking retail packaging that highlights a vibrant cherry red shade of the surface color [5]. Unfortunately, the use of vacuum packaging methods has been limited because rgw barrier properties of the film limit the interactions of the meat surface with oxygen and the conversion of surface colors that visually appear redder. However, it has been noticeable that a bright cherry-red can be achieved as a favorable surface color of ground beef even when placed in vacuum packaging [6]. 

Changes to the surface color of fresh and frozen meat are dependent on the concentration of meat pigments, the oxidation of these pigments, and physical characteristics such as light scattering [7]. Fresh meat color can be determined by the relative behavior of the three myoglobin derivatives [8]. Certain reduced forms of myoglobin such as metmyoglobin can result in a brown color that is often associated with the deterioration of fresh beef quality by consumers [7]. Identifying solutions to reduce meat color deterioration are of significant consideration throughout the global meat industry, as metmyoglobin concentrations exceeding 40% can exert a negative influence on consumer purchasing behavior [9]. Historically, the perception of fresh beef in vacuum pouches has often been perceived as a purplish red color, causing consumers to seek alternative protein choices due to surface color appearing brighter red. However, improvements in plastic film construction have created new methods for packaging fresh meat using vacuum packaging, though the foundational information regarding new packaging films on beef color is limited.

The deterioration of food quality can be attributed to lipid oxidation, which is facilitated by heme compounds [10]. A major cause of flavor deterioration in meat is the oxidation of unsaturated fatty acids [11]. Fatty acids in meat are composed mostly of triglycerides and phospholipids, which can be affected by the packaging method, leading to the storage stability of frozen meat [12]. A method often used to quantify lipid oxidation is the use of 2-thiobarbituric acid reactive substances (TBARS), resulting in malondialdehyde (MDA) equivalents, derived from tetraethoxypropane and identified as a by-product that occurs during the lipid oxidation process throughout the storage of fresh and cooked meats [13,14]. 

Packaging methods for fresh meat at the point of sale are undergoing changes, primarily influenced by a shift toward centrally packaged meats and a growing consumer demand for enhanced quality, safety, and convenience [15]. Vacuum-packaged meat has been conventionally employed to extend the freshness of beef over long-distance transportation and storage periods [16]. Vacuum packaging using thermoforming films involves enclosing a product in a package with or without barrier properties, then evacuating residual air to inhibit the growth of aerobic spoilage organisms, minimize shrinkage, prevent oxidation, and preserve color quality [17].

The objectives of the current study were to evaluate the lipid oxidation and surface color changes to vacuum-packaged ground beef bricks through 42 days of refrigerated storage under constant light exposure in retail cases, following 21 days of frozen storage.

## 2. Materials and Methods

### 2.1. Raw Materials

Beef chuck-eye rolls (USDA Institutional Meat Purchasing Specification #116A) were purchased from a commercial meat processing facility, transported to the Auburn University Lambert-Powell Meat Laboratory under refrigerated conditions 1.5 °C ± 0.5 °C, then stored for 24 h prior to grinding and packaging. At the time of grinding, coarse meat (227.27 kg) was allocated randomly to 1 of 3 treatments (N = 64.09 kg/treatment) and coarse ground once through a 9.525 mm plate (SPECO 400, Shiller Park, IL, USA) using a commercial meat grinder (Model AFMG-48, The Biro Manufacturing Company, Marblehead, OH, USA). Three batches (n = 21.36 kg/batch) of coarse ground beef per treatment were then ground once through a 3.18 mm plate (SPECO 400, Schiller Park, IL, USA). After grinding, ground beef was portioned into 454 g bricks using a vacuum stuffer (Model-VF608plus, Handtmann, Biberach, Germany). Ground beef bricks were stored in the absence of light at frozen temperatures −20.83 °C (±1.50 °C) for 21 days to simulate the logistical transportation of U.S. ground beef. Bricks were then transferred to refrigerated temperatures 3.0 °C ± 1.5 °C for instrumental color and lipid oxidation measurements to occur on days 0, 7, 14, 21, 28, 35, and 42 during refrigerated conditions. 

### 2.2. Packaging Treatments

The packages of ground beef (n = 47 bricks/batch) were sealed using a Variovac Optimus (OL0924, Variovac, Zarrentin am Schaalsee, Germany). Ground beef bricks were placed in one of three different thermoforming packaging films (T1, T2, and T3) and sealed with a standard non-forming layer constructed with the following parameters: 75 μ nylon/EVOH/enhanced/poloefin plastomer coextrusion with an oxygen transmission rate of 0.10 cc/sq. m/24 h and a vapor transmission rate of 4.0 g/sq. m/24 h, using commercial vacuum packaging procedures (WINKPAK, Winnipeg, MB, Canada). Packaging film specifications for vapor (VPR) and oxygen transmission rates (OTR) are presented in Table 1. The forming parameters of packaging film were conducted using 110 °C ± 1.5 °C of heat and 0.650 bar of pressure, and the sealing of packages was completed using 5 bar of vacuum and 135 °C ± 1.10 °C. After packaging, ground beef brick packages were individually labelled to identify their respective treatment and batch, placed into a cardboard box, and stored in the absence of light.

### 2.3. Simulated Storage Periods

Ground beef bricks were stored in the absence of light at −20.83 °C (±1.50 °C) for 21 days to simulate a frozen period of distribution. Bricks were placed into cardboard boxes and stored in a blast freezer (Model LHE6950, Larkin, Stone Mountain, GA, USA). At the conclusion of frozen storage, bricks were placed into a refrigerated (Day 0), multi-deck, lighted display case (Avantco, Model 178GDC49HCB, Turbo Air Inc., Long Beach, CA, USA), operating at 3.0 °C ± 1.5 °C. After 21 days of frozen storage, ground beef bricks were exposed under constant lighting for 42 days. Lighting within the case consisted of cool LED strips (TOM-600-12-v4-3, Philips Xitanium 40 W–75 W, Seoul, Korea) with a lighting intensity of 2297 lux (ILT10C, International Light Technologies, Peabody, MA, USA).

### 2.4. Lipid Oxidation

Throughout the 42 days of refrigerated storage, ground beef bricks were sampled (n = 5 bricks/batch/day) for 2-thiobarbituric acid-reactive substances (TBARS), as previously described [18]. Briefly, 2.0 g (±0.5) of ground beef was homogenized into a uniform sample in duplicate and mixed with 8 mL of cold (1 °C) 50 mM phosphate buffer (pH 7.0), containing 0.1% ethylenediamine-tetraacetic acid (EDTA), 0.1% n-propyl gallate, and 2 mL of trichloroacetic acid (Sigma-Aldrich, Saint Louis, MO, USA). Homogenized samples were filtered through a Whatman No. 1 filter paper into borosilicate glass tubes, and duplicate 2 mL aliquots of clear filtrate was transferred into 10 mL test tubes. Filtrate was mixed with 2 mL of 0.02 M 2-thiobarbituric acid reagent (BeanTown Chemical, Hudson, NH, USA) and placed into a hot water bath (100 °C) for 20 min. After the hot water bath, tubes were transferred to an ice bath for 15 min. The absorbance of each sample was measured at 533 nm with a spectrophotometer (VWR UV-1600 VWR International, Radnor, PA, USA) and multiplied using a factor of 12.21 to derive the TBARS value (mg of malonaldehyde/kg of fresh meat). The value of 12.21 was obtained previously from a standard curve using a known malonaldehyde solution measured across multiple absorbences [18].

### 2.5. Instrumental Color

Instrumental color was measured with a HunterLab MiniScan EZ colorimeter, Model 45/0 LAV (Hunter Associates Laboratory Inc., Reston, WV, USA), conforming to American Meat Science Association (AMSA) Meat Color Measurement Guidelines [19]. Surface color values were collected on 36 bricks/treatment (n = 12 bricks/batch/treatment) on days 0, 7, 14, 21, 28, 35, and 42 through the packaging film. Prior to surface color readings, the colorimeter was standardized using a black and white tile covered with the packaging films to confirm instrument accuracy. 

**Figure 1:**
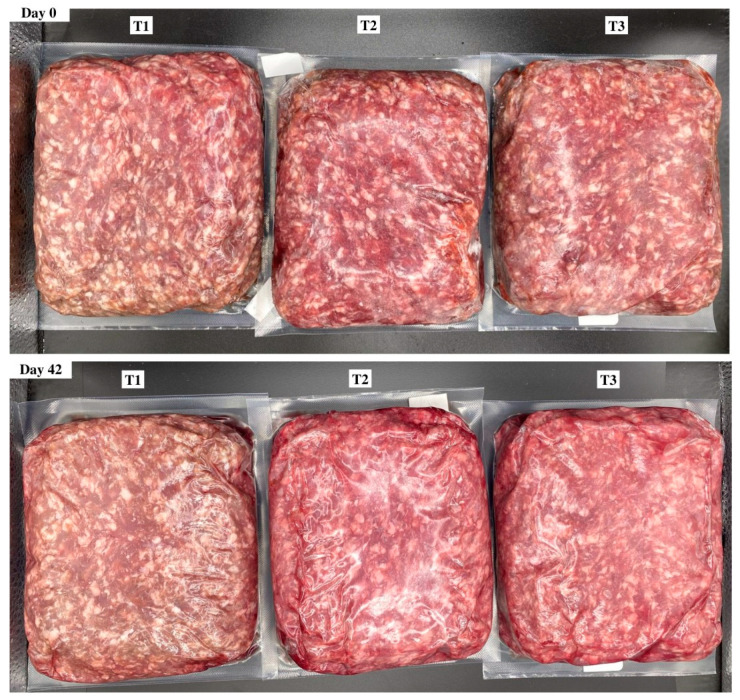
Surface color of ground beef during refrigerated retail display. Packaging treatments are defined as follows: T1 (150 µ polyethylene/EVOH/polyethylene coextrusion), T2 (175 µ polyethylene /EVOH/ polyethylene coextrusion), and T3 (200 µ polyethylene /EVOH/ /polyethylene coextrusion).

Objective color values were determined from the average of three readings per package using illuminant A, a 10° observer, and a 31.88 aperture for the lightness (L*), redness (a*), and yellowness (b*) of each brick. Furthermore, the calculated values of hue angle (°) were determined by tan−1 (b*/a*), and chroma (C*) was calculated using the √ a*^2^ + b*^2^. Reflectance values from 400 to 700 nm were used to record surface color changes from red to brown using the reflectance ratio of 630 nm:580 nm. In addition, the relative values of myoglobin redox forms such as deoxymyoglobin (%DMb = {2.375 × [1 − ({A473 − A700}/{A525 − A700})]} × 100), metmyoglobin (%MMb = {[1.395 − ({A572 − A700}/{A525 − A700})]} × 100), and oxymyoglobin (%OMb = 100 − (%MMb + %DMb) were calculated after measuring objective surface color readings using the handheld colorimeter. Delta E values indicated the total color change over a period and calculated as ∆E = [(∆L*)^2^ + (∆a*)^2^ + (∆b*)^2^]1/2; in addition, the ratios of a*:b* were calculated (a* ÷ b*), which indicate greater redness and less discoloration. Surface color measurements and the relative calculations of color data were conducted according to American Meat Science Association (AMSA) Meat Color Measurement Guidelines [19]. Visual surface color variation for packaging treatment and the day of storage are provided for reference (Figure 1). 

### 2.6. Proximate Analysis and pH Value

Using a near-infrared (NIR) approved spectrophotometer (Food Scan™, FOSS Analytical A/S, Hilleroed, Denmark) and data processing ISIscan™ Software (version 4.8, Höganäs, Sweden), one brick per batch was measured for proximate analysis (protein, moisture, and fat). Lastly, pH values were obtained by weighing 2 g of ground beef into a plastic centrifuge tube, adding 20 mL of deionized water, and homogenizing (Kinematica CH-6010, Brinkmann Instruments, Inc., Westbury, NY, USA) for 45 s. Afterwards, pH was measured using a pH meter (ModelHI99163, Hanna Instruments, Woonsocket, RI, USA) equipped with a glass electrode. The calibration of the pH meter was completed (pH 4.0 and pH 7.0) using 2-point standard buffers (Thermo Fisher Scientific, Chelmsford, MA, USA) prior to sampling. Mean values for proximate analysis and pH of ground beef within each treatment are presented for reference (Table 2). Results for proximate analysis are merely presented for the reference support of objective surface color measurements. 

### 2.7. Statistical Analysis

Data were analyzed as a completely randomized block design using the GLIMMIX model procedure of SAS (version 9.2; SAS Inst., Cary, NC, USA). Batch was included in the model as the random effect, and packaging treatment and day were the fixed effects. Least square means were computed for the variables, and significant (*p* ≤ 0.05) F-values were separated using a pair-wise *t*-test (PDIFF option).

## 3. Results and Discussion

### 3.1. Instrumental Color

After frozen storage for 21 days, ground beef bricks were stored in retail display cases, and color was analyzed objectively through the surface of the packaging film every 7 days for 42 days of refrigerated storage. There was no interaction of packaging treatment × storage day for lightness (*p* = 0.5925), redness (*p* = 0.0919), or yellowness (*p* = 0.8965) on the surface of the packaged ground beef (Table 3). A lack of interaction for the objective surface color suggests that the surface color of ground beef may have been protected from deterioration through the combination of colder storage temperatures and packaging technologies such as greater barriers reducing the OTR. Main effects for the lightness of fresh ground beef may have been influenced by the exposure to illuminated display affecting oxymyoglobin formation and the properties of the packaging film. 

Regardless of sampling day, ground beef stored in packaging treatments T1 and T3 was lighter (*p* = 0.0116) than ground beef packaged using T2 (Table 3), whereas redness values (a*) were greater (*p* < 0.0001) for T2 and T3. Current findings agree with previous research, where the objective redness values were greater in ground beef using vacuum packaging, in contrast to those obtained using either modified atmosphere packaging (MAP) or overwrap [20]. Similar to lightness, ground beef surface yellowness values were greater for T1 ground beef bricks (*p* < 0.0001). Changes in the surface color of the packages are likely attributed to the relationship of oxygen with the meat product, thereby accelerating the oxidation process. Previous research on ground beef tends to differ with the current findings. When identifying packaging with high and low oxygen transmission rates, few to no statistical differences for surface yellowness have been reported [20]. Variation in objective color for packaging treatments may be accredited to the elevated oxygen transmission rates associated with the packaging films of T1 and T2, allowing for greater concentrations of oxygen to pass through the barrier levels of the film to the surface of the meat or, conversely, due to a protective effect on the surface deterioration, as seen when using T3 by limiting oxygen exposure. 

Storage day greatly influenced ground beef lightness (*p* < 0.0001), with bricks appearing darkest on day 0 and lightest on day 35 (Table 3). Previous research has reported that surface lightness is greatest after only 14 days of evaluation [21]. Ground beef bricks were redder (*p* < 0.0001) on days 14 and 21, but darkest on day 0. The current results of redness agree with those previously reported using a traditional PVC package on ground beef, where the highest point in this parameter occurred at 14 days [21]. Yellowness values (b*) increased from day 14 (*p* < 0.0001), which contrasts with another study that evaluated the stability of ground beef in traditional packaging with regard to storage duration, which reported a decline on day 28 [21].

The surface redness of beef products during retail storage has been instrumental in altering consumer purchasing. The relative spectral values of redness calculated as hue angle and red-to-brown offer another resource to evaluate surface color changes that are specific to redness. Calculated spectral redness for ground beef bricks did not result in a packaging treatment × day of storage interaction for hue angle (*p* = 0.3306) or red-to-brown (*p* = 0.4393). However, significant impacts on main effects for the packaging treatment and storage day of the ground beef did occur (Table 4). 

Hue angle represents the objective progression of surface color from red to yellow, with greater angles as a measure of declining redness. Ground beef placed in T1 packaging treatment had greater hue angle values (*p* < 0.0001) than ground beef in treatments T2 or T3. It is likely that the greater hue angle for T1 can be attributed to the greater oxygen permeability of the packaging film, allowing for a greater association of oxygen with the surface of ground beef during storage. Comparable hue angles were reported in a study focused on the color of beef from mature cows during display using a high-oxygen-atmosphere package, which reported that hue angle values were greater as oxygen levels increased [22]. Additionally, throughout the storage time, hue angle was greatest (*p* < 0.0001) on day 7 and again at day 42, which agrees with previous research, in which values increased after 5 days of storage [23]. It has been documented that prolonged frozen storage leads to increased discoloration and a decrease in redness, consistent with the penetration of oxygen [22]. Current results suggest that frozen storage prior to refrigerated display contributes significantly to meat surface color changes [22]; without a doubt, more research is needed to identify these changes that occur during different storage temperature when using thermoforming vacuum packaging. 

Red-to-brown measurement was obtained by calculating the ratio reflectance at 630:580 nm from the spectral values; this is also frequently used to determine the surface redness of meat. In contrast to hue angle measurements, ground beef packages in T2 and T3 were redder (*p* < 0.0001) than ground beef in T1. It appears, based on the surface color differences, that a greater percentage of oxygen was able to pass through T1 layers within the packaging film, and it is likely that the protective barriers of T2 and T3 caused less dissociation of oxygen and preserved the red surface color. Current results suggest that ground beef packages in thermoforming appear to have a greater impact on red-to-brown than those previously reported in a study that evaluated the color stability of ground beef packaged in a low-carbon-monoxide atmosphere [23]. Storage day influences on red-to-brown values (*p* < 0.0001) increased through 14 days of storage (Table 4) and then declined through the remaining 28. This increasing action and subsequent decline contradict what has been reported previously, which is that the red-to-brown decline occurs immediately on the first day of storage [24].

**Figure 2:**
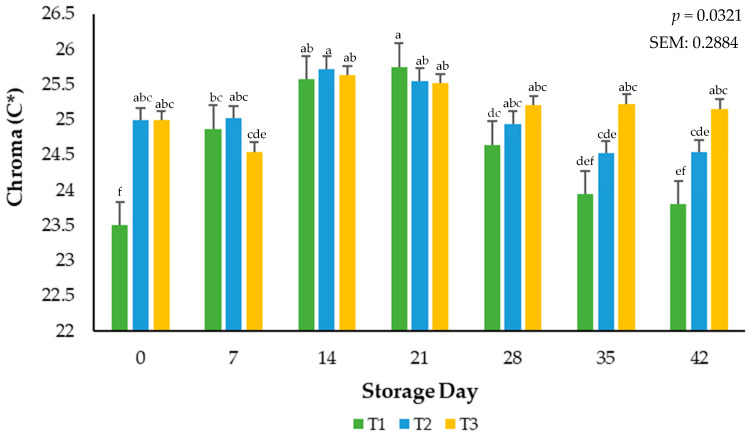
Calculated Chroma values for the interactive impact of packaging method × storage day. C* (Chroma) is a measure of total color (a larger number indicates a more vivid color) ^a–f^ Mean values within a color measurement lacking common superscripts differ (*p* < 0.05). SEM: standard error of the mean.

Surface color vividness is a result of calculated relative spectral values and is reported as Chroma. An interaction (*p* = 0.0321) of packaging treatment × storage day occurred during the current study (Figure 2). During the evaluation period, treatment T1 demonstrated the highest saturation point by day 21, contrasted with treatment T2, which exhibited a gradual decrease in values throughout the time of evaluation. However, ground beef stored in the T3 packaging film showcased a comparatively stable development as opposed to the other treatments, particularly notable at the conclusion of the 42-day assessment period. Similar saturation index performance was observed in a study where the shelf-life and stability of ground beef packaged in a traditional overwrap for 28 days were evaluated [21].

The relative values of myoglobin forms were calculated using objective measurements obtained throughout the storage period. There was no significant interaction between packaging treatment × storage duration for metmyoglobin (*p* = 0.2810), deoxymyoglobin (*p* = 0.2284), or oxymyoglobin (*p* = 0.10339). However, main effects for each calculated relative value of myoglobin recorded from objective spectral values are presented for treatment and the days of storage in Table 5. 

Packaging treatment caused an effect on the calculated relative values of myoglobin (Table 5). The packaging of ground beef in T2 and T3 resulted in less (*p* < 0.0001) calculated metmyoglobin, in contrast with treatment T1. However, packaging treatment (T3) resulted in the greatest relative value of oxymyoglobin (OMb) during storage (*p* = 0.0460). Vacuum packaging of fresh meat has been criticized for limiting surface color changes during storage. The current results of objective surface color for relative myoglobin forms indicate that OTR and VPR are influential in the transition of surface color. Packaging film T1 was constructed with fewer barrier properties associated with the altered forms of myoglobin. The altered surface color of myoglobin forms could ultimately change consumer perceptions of surface color. Specifically, previous research has revealed a markedly diminished level of metmyoglobin and deoxymyoglobin content when using vacuum packaging, but an inverse relationship with the amount of relative oxymyoglobin [25]. 

The day of storage also contributed to changes in the calculated relative forms of myoglobin within the packaged ground beef (Table 5). Main effects differed for metmyoglobin values and were greater upon removal from frozen storage on day 0 (*p* < 0.0001) and then declined by more than 45% through the first 7 days of refrigerated storage. Surprisingly, oxymyoglobin values were greatest (*p* < 0.0001) on day 7 of storage and remained relatively greater than expected during a prolonged storage period of 42 days. Packaging technology is improving, as noted by the estimated values of relative myoglobin, specifically oxymyoglobin. Oxymyoglobin has been historically referenced for influencing the consumer purchasing of fresh meat because it is associated with a redder surface color. Current results suggest that packaging films such as thermoforming can protect the surface color variables and that the film OTR is influential in changes that occur to fresh meat color. Differences in the relative values of met- and oxymyoglobin agree with previous research when using vacuum packaging methods for storing retail beef loin cuts, but current results different for deoxymyoglobin levels, which reported increasing calculated values of deoxymyoglobin during storage [26]. 

### 3.2. Calculated Relative Pigments

**Figure 3:**
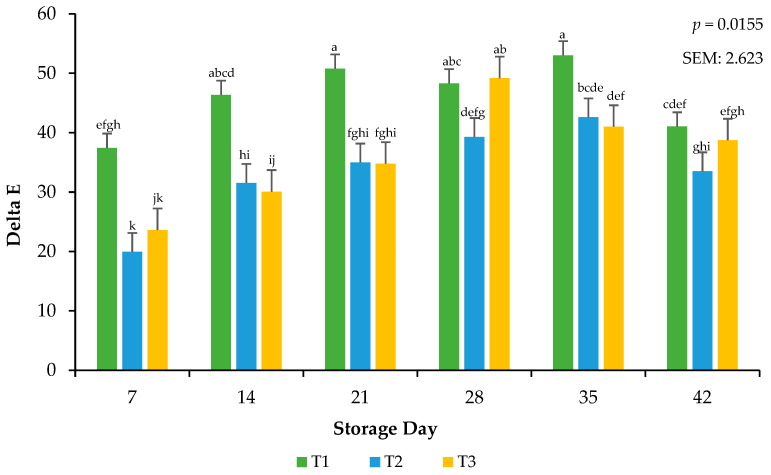
Calculated Delta E values for the interactive impact of packaging method × storage day. Delta E: Total color change over storage time. ^a–k^ Bars lacking a common superscript differ (*p* < 0.05). SEM: standard error of the mean.

Delta E was calculated from objective color readings to assess the total color change that occurred on the meat surface during the storage period [19]. Calculations for relative pigments aid in supporting the traditional values of L*, a*, and b*. Because the surface color was exposed to frozen temperatures, it was possible that color deterioration could have occurred more rapidly in the current study. Delta E was used to confirm that, even if color change was minimal, as recorded by the colorimeter, the change could be visualized. There was an interactive effect for the packaging method × day of storage on calculated delta E (*p* = 0.0155; Figure 3). Ground beef packaged in T1 had greater surface color changes throughout storage than ground beef packaged in T2 and T3, an effect that may be due to the high permeability (lower OTR and VPR) that characterizes these treatments, as a high permeability tends to promote a slower color change compared to packaging with very low permeability [22]. Current findings are consistent with previous research, which has reported that the extended frozen storage of beef steaks in packaging with varying permeability levels leads to increased surface color changes, attributed to reduced metmyoglobin activity and resulting in greater discoloration over time [27]. More research needs to be conducted that evaluates the total color changes that occur during storage and the mechanisms aside from packaging film oxygen transmission rate that cause these changes. 

**Figure 4:**
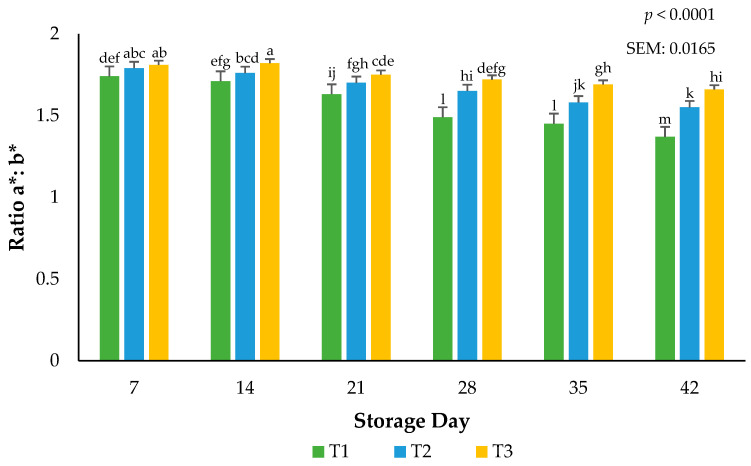
Calculated ratios of a*:b* for the interactive impact of packaging method × storage day. Larger ratios indicate more redness and less discoloration. ^a–m^ Bars lacking a common superscript differ (*p* < 0.05). SEM: standard error of the mean.

Larger a*:b* ratios indicate more redness and less discoloration [19]. Focusing only on surface redness (a*) may impede our understanding of the overall surface color changes that are linked to the hues of red. Surface redness in fresh meat such as beef, pork, and lamb has been well supported in the literature as to their influence on consumer purchasing intent. The current use of calculated ratios for redness supports the findings in this submission that packaging barriers are instrumental in stabilizing the red hues of beef, even when altered during frozen storage. An interaction between packaging film and storage day for the ratios of a*:b* values occurred (*p* < 0.0001). Ground beef packaged in T1 had more discoloration than ground beef packaged using T2 or T3 films (*p* < 0.0001), indicating that the barrier properties of the packaging films can accelerate or stabilize surface color changes (Figure 4). Greater a*:b* ratio values can be adjudicating to the packaging permeability of atmospheric gases such as oxygen [19]. Protecting surface color during storage is paramount to ensuring consumer acceptance at the time of purchase. These results agree with previous research where the surface discoloration of beef steaks utilizing low permeability packaging increased when extended storage duration occurred [21]. A rise in the use of vacuum packaging in the U.S. at the retail counter suggests agreement with the current results that ground beef can withstand temperature and excessive storage duration [3].

### 3.3. Lipid Oxidation 

**Figure 5:**
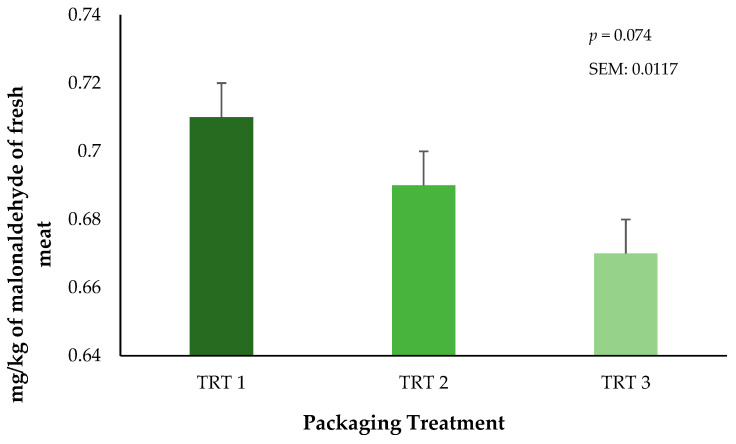
Influence of packaging film treatments on the lipid oxidation values of ground beef bricks. Packaging treatments are defined as follows: T1 (150 µ polyethylene/EVOH/polyethylene coextrusion), T2 (175 µ polyethylene /EVOH/ polyethylene coextrusion), and T3 (200 µ polyethylene /EVOH/ /polyethylene coextrusion).

Lipid oxidation was measured through the quantification of malonaldehyde (MDA) within the fresh ground beef throughout the storage periods. There was no interaction of treatment × day on lipid oxidation in ground beef during the current study (*p* = 0.4104, results not presented). The main effect of packaging treatment did not alter lipid oxidation (*p* = 0.0744) of the ground beef bricks (Figure 5). These results suggest that the OTR of the vacuum packaging film plays a significant role in the inhibition of the lipid oxidation in ground beef regardless of the combined storage duration that occurred in frozen and fresh conditions. Unfortunately, lipid oxidation was not measured during the 21 days of frozen storage, but it is possible that the storage of meat products at low temperatures can reduce oxidative effects when they are protected by a package [28]. During fresh storage, packaging treatment T3 had numerically lower TBAR values during storage. This trend is likely attributed to the greater barrier properties of this film, which would be expected to reduce OTR and VPR. Current results agree with prior research on beef cuts with different packaging methods, where authors have reported that using vacuum packaging generates lesser TBAR values compared to beef cuts stored using either wrapped or CO_2_ packaging methods [29]. Nevertheless, additional research is needed to identify the growth of specific microorganisms that occur during the storage of beef products stored in thermoforming packaging and the subsequent association to lipid oxidation. However, the objectives of the current research were aimed at only surface color and lipid oxidation characteristics that occurred during frozen storage prior to refrigerated storage. 

**Figure 6:**
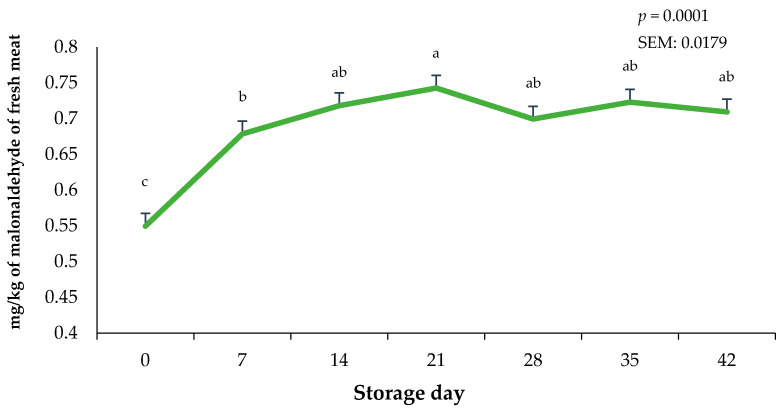
Impact of storage day on 2-thiobarbituric acid-reactive substances on ground beef bricks during 42 days of refrigerated storage. ^a–c^ Bars lacking a common superscript differ (*p* < 0.05). SEM: standard error of the mean.

The storage duration of vacuum-packaged ground beef significantly altered lipid oxidation values (*p* < 0.0001). Lipid oxidation in the bricks of ground beef were greatest on day 21 (*p* < 0.0001) and least on day 0 of the storage period (Figure 6). Interestingly, regardless of the 21-day frozen storage and the 42-day fresh combined storage duration, lipid oxidation did not exceed 1.0 mg of malonaldehyde, which has been previously linked to detectable oxidation flavors by consumers [30]. Changes in lipid oxidation likely occurred due to the combination of case lighting causing the rapid deterioration of fat in the ground beef bricks and the increased storage temperature when bricks were moved from −20 °C to 3.0 °C. In contrast, whole muscle using packaging films offering OTR protection can likely reduce TBAR values even more than in the current study [29]. Previous research on the lipid oxidation potential of beef, chicken, and pork has indicated that raw red meats are more prone to lipid oxidation due to a greater presence of heme pigments [31]. It has also been reported that lipid oxidation values in frozen raw muscles were greater for beef compared to pork or chicken, suggesting that myoglobin acts as a major catalyst for lipid oxidation during storage [31]. 

Current findings for TBARS align with some studies that suggest that the oxidative behavior of beef increases from the first day of exposure to constant lighting within the retail case and grows larger as storage duration increases [9]. Likewise, extending storage periods to greater than 21 days has been associated with increased lipid oxidation in ground beef patties [32]. Nevertheless, present findings suggest that, after a combined 21 days of dark frozen storage and 42 days of fresh storage with exposure to retail case lighting, malonaldehyde (MDA) levels remain below the range detectable by consumers, as rancid taste in beef has been documented to be noticeable above 1.0 mg malonaldehyde/kg tissue [33].

## 4. Conclusions

Storing ground beef at frozen temperatures prior to refrigeration and fresh display when using thermoforming vacuum packaging did not cause disruption to lipid oxidation. However, storage duration demonstrated that lipid oxidation will increase, yet vacuum packaging will allow for lipid oxidation to remain within acceptable thresholds, even after 63 days of total storage. The barrier components of the packaging films can stabilize surface color attributes from rapid deterioration normally observed in aerobic packaging such as poly-vinyl chloride film or even modified atmosphere. Thermoforming is a promising new packaging platform used for consumer retail meats. However, changes to packaging film properties that alter the interaction of meat proteins with atmospheric gases, leading to changes in surface color, suggests that more results are needed. Nevertheless, more research should prioritize the investigation of microbial populations and sensory taste attributes when extended storage conditions are considered, regardless of frozen or fresh temperatures. 

## Figures and Tables

**Table 1:** Vacuum packaging components and treatment allocation for thermoforming and non-forming films.

Treatment	Components ^1^	OTR ^2^	VPR ^3^
T1	150 µ polyethylene/EVOH/polyethylene coextrusion	0.6 cc/sq. m/24 h	3.2 g/sq. m/24 h
T2	175 µ polyethylene /EVOH/ polyethylene coextrusion	0.5 cc/sq. m/24 h	2.8 g/sq. m/24 h
T3	200 µ polyethylene /EVOH/ /polyethylene coextrusion	0.4 cc/sq. m/24 h	2.4 g/sq. m/24 h

^1^ Packaging treatment composition. ^2^ OTR: Oxygen transmission rates. ^3^ VPR: Vapor transmission rates.

**Table 2:** Relative mean values for proximate analysis and ultimate pH of ground beef.

	Packaging Treatments ^1^		
	T1	T2	T3
pH	5.84	5.80	5.79
Protein (%)	22.86	22.62	22.47
Fat (%)	15.20	15.22	15.62
Moisture (%)	68.62	68.49	68.16

^1^ Packaging treatments are defined as follows: T1 (150 µ polyethylene/EVOH/polyethylene coextrusion), T2 (175 µ polyethylene /EVOH/ polyethylene coextrusion), and T3 (200 µ polyethylene /EVOH/ /polyethylene coextrusion).

**Table 3:** Influence of packaging film treatments on the instrumental surface color of ground beef.

	Surface Color Parameters ^1^		
	Lightness (L*)	Redness (a*)	Yellowness (b*)
Packaging Treatment ^2^			
T1	47.87 ^a^	20.46 ^b^	13.51 ^a^
T2	47.24 ^b^	21.44 ^a^	12.90 ^b^
T3	47.70 ^a^	21.76 ^a^	12.65 ^b^
Storage Day ^3^			
0	40.92 ^Z^	20.29 ^Y^	13.49 ^V^
7	46.88 ^Y^	21.62 ^VW^	12.17 ^Y^
14	48.22 ^X^	22.29 ^V^	12.66 ^X^
21	48.91 ^W^	22.02 ^V^	13.06 ^WX^
28	49.70 ^V^	21.19 ^WX^	13.10 ^VW^
35	49.74 ^V^	20.69 ^XY^	13.20 ^VW^
42	48.84 ^WX^	20.44 ^XY^	13.45 ^VW^
*p*-value (Day) *	<0.0001	<0.0001	<0.0001
*p*-value (Treatment) **	0.0116	<0.0001	<0.0001
*p*-value (Day × Treatment) ***	0.5925	0.0919	0.8965
SEM (Day) *	0.223	0.284	0.150
SEM (Treatment) **	0.146	0.186	0.098

^1^ Surface color parameters: L* values are a measure of darkness to lightness (a larger value indicates a lighter color); a* values are a measure of redness (a larger value indicates a redder color); and b* values are a measure of yellowness (a larger value indicates a more yellow color). ^2^ Packaging treatments are defined as follows: T1 (150 µ polyethylene/EVOH/polyethylene coextrusion), T2 (175 µ polyethylene /EVOH/ polyethylene coextrusion), and T3 (200 µ polyethylene /EVOH/ /polyethylene coextrusion). ^3^ Storage day refers to 42 days of refrigerated storage with constant light exposure in the retail cases following 21 days of frozen storage. ^a,b^ Mean values within the main effect of treatment for color measurements lacking common superscripts differ (*p* < 0.05). ^V–Z^ Mean values within the main effect of day for color measurement lacking common superscripts differ (*p* < 0.05). *p*-value *: packaging treatment main effect, *p*-value **: effect of storage day main effect, and *p*-value ***: packaging treatment × storage day interaction. SEM *: standard error of the mean for packaging treatment, SEM **: standard error of the mean for storage day.

**Table 4:** Impact of packaging film on the calculated relative spectral values of ground beef.

	Calculated Relative Spectral Parameters ^1^	
	Hue Angle (°)	Red-to-Brown (RTB)
Packaging Treatment ^2^		
T1	33.61 ^a^	2.59 ^b^
T2	31.07 ^b^	2.83 ^a^
T3	30.19 ^b^	2.91 ^a^
Storage Day ^3^		
0	33.90 ^W^	2.80 ^Y^
7	29.38 ^Z^	3.13 ^W^
14	29.61 ^Z^	3.01 ^WX^
21	30.67 ^YZ^	2.88 ^XY^
28	31.75 ^XY^	2.59 ^Z^
35	32.6 ^WX^	2.52 ^Z^
42	33.90 ^W^	2.49 ^Z^
*p*-value (Day) *	<0.0001	<0.0001
*p*-value (Treatment) **	<0.0001	<0.0001
*p*-value (Day × Treatment) ***	0.3306	0.4393
SEM (Day) *	0.658	0.069
SEM (Treatment) **	0.431	0.045

^1^ Calculated relative spectral parameter refers to hue angle (°), representing the change in color from the true red axis (a larger number indicates a greater shift from red to yellow); RTB is the reflectance ratio of 630 nm ÷ 580 nm and represents a change in the color from red to brown (a larger value indicates a redder color). ^2^ TRT: packaging treatments are defined as follows—T1 (150 µ polyethylene/EVOH/polyethylene coextrusion), T2 (175 µ polyethylene /EVOH/ polyethylene coextrusion), and T3 (200 µ polyethylene /EVOH/ /polyethylene coextrusion). ^3^ Storage day refers to 42 days of refrigerated storage with constant light exposure in the retail cases following 21 days of frozen storage. ^a,b^ Mean values within the main effect of treatment for color measurements lacking common superscripts differ (*p* < 0.05). ^W–Z^ Mean values within the main effect of day for color measurement lacking common superscripts differ (*p* < 0.05). *p*-value *: packaging treatment main effect, *p*-value **: effect of storage day main effect, and *p*-value ***: packaging treatment × storage day interaction. SEM *: standard error of the mean for packaging treatment, SEM **: standard error of the mean for storage day.

**Table 5:** Calculated relative spectral values for the packaging treatment effect on the myoglobin forms of ground beef.

Relative Myoglobin Forms ^1^			
	Metmyoglobin (MMb)	Deoxymyoglobin (DMb)	Oxymyoglobin (OMb)
Packaging Treatment ^2^			
T1	24.49 ^a^	29.06 ^c^	46.45 ^b^
T2	21.22 ^b^	31.25 ^b^	47.52 ^a^
T3	20.00 ^b^	32.72 ^a^	47.28 ^ab^
Storage Day ^3^			
0	29.65 ^U^	22.51 ^Z^	47.84 ^V^
7	16.30 ^W^	33.26 ^VW^	50.44 ^U^
14	17.85 ^W^	35.14 ^U^	47.02 ^V^
21	18.68 ^W^	34.15 ^UV^	47.18 ^V^
28	23.00 ^V^	31.81 ^WX^	45.20 ^W^
35	23.97 ^V^	30.63 ^XY^	45.40 ^W^
42	23.90 ^V^	29.58 ^Y^	46.53 ^VW^
*p*-value (Day) *	<0.0001	<0.0001	<0.0001
*p*-value (Treatment) **	<0.0001	<0.0001	0.0460
*p*-value (Day × Treatment) ***	0.2810	0.2284	0.1033
SEM (Day) *	1.036	0.617	0.470
SEM (Treatment) **	0.678	0.404	0.308

^1^ Calculated relative spectral values for myoglobin forms: metmyoglobin (MMb), deoxymyoglobin (DMb), and oxymyoglobin (OMb). ^2^ TRT: packaging treatments are defined as follows: T1 (150 µ polyethylene/EVOH/polyethylene coextrusion), T2 (175 µ polyethylene /EVOH/ polyethylene coextrusion), and T3 (200 µ polyethylene /EVOH/ /polyethylene coextrusion). ^3^ Storage Day: refers to 42-days of refrigerated storage with constant light exposure in retail cases following 21 days of frozen storage. ^a–c^ Mean values within the main effect of treatment for color measurements lacking common superscripts differ (*p* < 0.05). ^U–Z^ Mean values within the main effect of day for color measurement lacking common superscripts differ (*p* < 0.05). *p*-value *: packaging treatment effect, *p*-value **: effect of storage day, and *p*-value ***: packaging treatment × storage day interaction. SEM *: standard error of the mean for packaging treatment, SEM **: standard error of the mean for storage day.

## Data Availability

The original contributions presented in the study are included in the article, further inquiries can be directed to the corresponding author.
